# Primary HIV infection features colonic damage and neutrophil inflammation yet containment of microbial translocation

**DOI:** 10.1097/QAD.0000000000003799

**Published:** 2023-12-04

**Authors:** Camilla Tincati, Valeria Bono, Elvira Stefania Cannizzo, Delfina Tosi, Federica Savi, Camilla Falcinella, Anna Casabianca, Chiara Orlandi, Carmelo Luigiano, Matteo Augello, Stefano Rusconi, Antonio Muscatello, Alessandra Bandera, Andrea Calcagno, Andrea Gori, Silvia Nozza, Giulia Marchetti

**Affiliations:** a Clinic of Infectious Diseases, Department of Health Sciences, University of Milan; b Pathology Unit, Department of Health Sciences, ASST Santi Paolo e Carlo, University of Milan, Milan; c Department of Biomolecular Sciences, University of Urbino Carlo Bo, Fano; d Digestive Endoscopy Unit, ASST Santi Paolo e Carlo; e UOC Malattie Infettive, Ospedale Civile di Legnano, Department of Biomedical and Clinical Biosciences, University of Milan; f Infectious Diseases Unit, Fondazione IRCCS Ca’ Granda Ospedale Maggiore Policlinico, Milan; g Unit of Infectious Diseases Unit, Department of Medical Sciences, University of Turin, Turin; h Clinic of Infectious Diseases, Department of Pathophysiology and Transplantation, ASST Fatebenefratelli Sacco University of Milan; i Infectious Diseases Unit, IRCCS Ospedale San Raffaele, Milan, Italy.

**Keywords:** gut barrier, microbial translocation, mucosal immunity, primary HIV infection

## Abstract

**Introduction:**

Impairment of the gastrointestinal barrier leads to microbial translocation and peripheral immune activation, which are linked to disease progression. Data in the setting of primary HIV/SIV infection suggest that gut barrier damage is one of the first events of the pathogenic cascade, preceding mucosal immune dysfunction and microbial translocation. We assessed gut structure and immunity as well as microbial translocation in acutely and chronically-infected, combination antiretroviral therapy (cART)-naive individuals.

**Methods:**

Fifteen people with primary HIV infection (P-HIV) and 13 with chronic HIV infection (C-HIV) c-ART-naive participants were cross-sectionally studied. Gut biopsies were analysed in terms of gut reservoirs (total, integrated and unintegrated HIV DNA); tight junction proteins (E-cadherin, Zonula Occludens-1), CD4^+^ expression, neutrophil myeloperoxidase (histochemical staining); collagen deposition (Masson staining). Flow cytometry was used to assess γδ T-cell frequency (CD3^+^panγδ+Vδ1+/Vδ2+). In plasma, we measured microbial translocation (LPS, sCD14, EndoCAb) and gut barrier function (I-FABP) markers (ELISA).

**Results:**

P-HIV displayed significantly higher tissue HIV DNA, yet neutrophil infiltration and collagen deposition in the gut were similar in the two groups. In contrast, microbial translocation markers were significantly lower in P-HIV compared with C-HIV. A trend to higher mucosal E-cadherin, and gut γδ T-cells was also observed in P-HIV.

**Conclusion:**

Early HIV infection features higher HIV DNA in the gut, yet comparable mucosal alterations to those observed in chronic infection. In contrast, microbial translocation is contained in primary HIV infection, likely because of a partial preservation of E-cadherin and mucosal immune subsets, namely γδ T-cells.

## Introduction

The damage of the gastrointestinal barrier is a key event in the pathogenesis of HIV/SIV infection and arises by different mechanisms.

HIV directly impairs the epithelial barrier [1] and depletes, aside from CD4^+^ T cells, a wide range of IL-17- and IL-22-producing mucosal cell subsets [2], which contribute to intestinal integrity and restrict commensal bacteria from the systemic circulation. Taken together, these events lead to mucosal impairment [3,4], microbial translocation [5] and peripheral inflammation [4], which are strictly linked to disease progression [6–8] as well as poor CD4^+^ T-cell recovery on combination antiretroviral therapy (cART) [9–14].

Given the critical role of the gastrointestinal tract in HIV infection, understanding the precise timing of the phenomena taking place therein is crucial for the elaboration and timely administration of adjuvant therapeutic interventions, which can counteract the key events in the HIV-driven pathogenic cascade.

Older work in acute HIV/SIV infection demonstrated rapid CD4^+^ T-cell loss at mucosal sites during the very first phases of infection [15,16], while little/no microbial translocation was shown in primary HIV infection [5,17]. In keeping with these findings, a study in the animal model demonstrated that proteins involved in epithelial integrity are altered 3 day post-SIV infection and precede Th17 depletion, which occurs approximately 2–3 weeks later [18], confirming previous data on impairment of mucosal Th17 frequencies and function no earlier than Fiebig stage III [19]. Recent work in acutely infected rhesus macaques showed that proteome changes linked to the transition of epithelial to mesenchymal cells in the gut occur before the mucosal antiviral response [20].

In untreated primary (P-HIV) and chronic (C-HIV) people living with HIV, we assessed the gut mucosa in terms of viral reservoir, mucosal fibrosis/inflammation and immunity as well as barrier structure. We also measured microbial translocation and gut damage markers in the peripheral blood.

## Methods

### Study design and population

This is a cross-sectional study enrolling people living with HIV in care at the Clinic of Infectious Diseases, University of Milan, ASST Santi Paolo e Carlo, Milan, Italy. Patients with Primary HIV (P-HIV; *n* = 15) infection were classified based on a positive p24 antigenemia or detectable HIV RNA with a negative or indeterminate Western Blot confirmation assay, according to Fiebig stage [21]. Individuals with chronic HIV infection (C-HIV, *n* = 13) were also included. Both P-HIV and C-HIV were naive to cART. Individuals with a known gastrointestinal disease or clinical symptoms were excluded from the study. Blood samples and intestinal biopsies during colonoscopy were obtained following the provision of informed consent, which was approved by the Institutional Review Board at the ASST Santi Paolo e Carlo, Milan, Italy. Experiments were carried out by the Laboratory of the Clinic of Infectious Diseases and by the Pathology Unit, Ospedale San Paolo, University of Milan, Italy.

### Colonoscopy

Patients underwent routine screening colonoscopy [bowel preparation: Moviprep (Norgine, Marburg, Germany); pretreatment: midazolam (2 mg) and pethidine (50 mg)]. Four pinch biopsies were collected in the colon: two biopsies were immediately transported to the Pathology Unit for cryopreservation with Optimal Cutting temperature Compound (OCT) and the other two were processed for flow cytometric analysis. Following this procedure, all colonic tracts, rectum and distal ileum were biopsied (one pinch biopsy per site) were formalin-fixed (10%) and processed for routine histopathological examination; one colonic biopsy was selected for immunohistochemical staining (see below).

### Immunohistochemical staining

One paraffin-embedded biopsy from transverse colon was selected, stained with haematoxylin–eosin (HE), Masson trichrome and antibodies against the following markers: major structural proteins of Tight Junction (TJs) [Zonula Occludens 1 (ZO-1, 1 : 200, Zymed) and Cadherin 1 (Cdh1, 1 : 15 000, ABNOVA)]; CD4^+^ (clone 1F6, 1 : 50; Leica Microsystems); CD8^+^ (clone C8/144B Mouse, Dako) neutrophil infiltration: myeloperoxidase (MPO) (rabbit antihuman MPO, Dako). The Autostainer Dako Omnis was used for the staining.

Expression of Tight Junction proteins in colonic epithelium of study participants was evaluated as compared with controls, that is, *n* = five surgical resection margins of colonic tissue removed for neoplastic disorder and expressed as follows: ZO1: focal, partial or total reduction with respect to the normal localization on the apical part of the cell membrane in three high-power fields (HPF); CDh1: reduction considered as expression only in some parts of the cell membrane (baso-lateral, lateral, basal) compared with normal localization on the entire perimeter of the membrane in three HPF. CD4^+^ T-lymphocytes were determined by number of CD4^+^ cells in three HPF of gut lamina propria.

### Masson staining

Collagen deposition in the gut was measured through Masson staining on formalin-fixed and paraffin-embedded sections (Bio-Optica Milano Spa). A semiquantitative score was used (0 = no staining, 1 = thin collagen fibre staining, 2 = thick collagen fibre staining, 3 = intense blue bundles staining).

### Flow cytometry

Lamina Propria (LP) mononuclear cells were extracted from intestinal biopsies using previously described techniques [22]. Tissue was rinsed with Hanks’ Balanced Salt Solution (HBSS, Cellgro, Manassas, Virginia, USA), then digested with 1–2 mg/ml of collagenase D (Roche, Nutley, New Jersey, USA) in RPMI containing 1% penicillin, 1% streptomycin and 1% glutamine (complete RPMI) supplemented with 0.1% BSA for one to two 60 min treatments. Released LP mononuclear cells (LPMC) from each treatment were passed through a cell strainer. All released cells ultimately pooled were utilized to perform flow cytometry. We evaluated frequencies of γδ T cells (CD3+panγδ+Vδ1+/Vδ2+) on intestinal biopsies. The following antibodies were used: anti-CD3-APC-H7, anti-TCR γδ-FITC, anti-Vδ1-APC, anti-Vδ2-PE, (BD Biosciences, La Jolla, California, USA). LIVE/DEAD Viability Dye (Thermo Fisher, Carlsbad, California, USA) was used to exclude dead cells. Samples were acquired with the Flow Cytometer FACSVerse (Becton Dickinson Italia Spa, Milan, Italy). FCS files were then analysed with FlowJo software.

Γδ T-cell subsets were also measured in colon samples from representative HIV-uninfected controls.

The gating strategy for γδ T cells is shown in Supplemental Figure 1.

### Markers of microbial translocation

Plasma sCD14, endotoxin core antibodies (EndoCAb) and intestinal FABP (I-FABP) were measured by Sandwich Enzyme Linked Immunosorbent Assay (ELISA) (sCD14: R&D Systems, Minneapolis, Minnesota, USA; minimum detectable dose (MDD) of human sCD14 less than 25 pg/ml; EndoCAb and I-FABP: HyCult Biotech, Uden, Netherlands; EndoCAb: MMD: 0.13 GMU/ml; I-FABP: MDD: 47 pg/ml) as per the manufacturers’ instructions. Circulating lipopolysaccharide (LPS) was assessed using the limulus amoebocyte lysate (LAL) test (Lonza Group L.T.D., Basel, Switzerland; labelled Lysate Sensitivity: 0.125 EU/ml), in accordance with the manufacturer's instructions.

### Total, unintegrated, and integrated HIV DNA quantification

Cellular DNA was isolated from gut biopsies with the protocol for DNA Purification from Tissues (QIAGEN QIAamp DNA Mini Kit) following manufacturers’ instructions. Total and unintegrated HIV-DNA forms were simultaneously analysed by *TotUFsys* qPCR platform using a single set of specific primers selected in the 5′ LTR-Gag highly conserved region of HIV-1 genome, as described by Casabianca *et al.*[23]. All PCR reactions were carried out in a 7500 Real-Time PCR system (Applied Biosystems, Thermo Fisher Scientific Inc., Carlsbad, California, USA) in a final volume of 100 μl using the Hot-Rescue Real-Time PCR Kit Sybr Green [Diatheva s.r.l., Cartoceto (PU), Italy] [24] assay testing 0.5 μg (at least in duplicate) and 1.0 μg of cellular DNA. The HIV-1 DNA copy number was estimated by interpolation of the experimentally determined threshold cycle (*C*_t_) on the standard curve (generated from 10^5^ to 10, and 2-copy numbers). Values less than two copies were arbitrarily considered to be 1 for statistical analyses. The amount of integrated HIV DNA was obtained by subtracting the amount of uDNA from the amount of total HIV DNA (tDNA). Total/unintegrated/integrated HIV DNA copy numbers were normalized (as described in [23–29]) to 1 μg of cellular DNA (equivalent to 142 857 cells) [30].

### Statistical analysis

Descriptive and statistical analyses were performed with GraphPad Prism 5.1 (GraphPad Inc., La Jolla, California, USA). Categorical variables are presented as number of cases and percentages, continuous variables are presented as median values and interquartile range. Categorical variables were analysed by two-sided Fisher's exact test, continuous variables were analysed by nonparametric two-tailed Mann–Whitney test or Kruskal–Wallis test wherever appropriate. Spearman's correlation test was used to correlate peripheral biomarkers and gastrointestinal variables. A *P* value less than 0.05 was considered statistically significant.

## Results

### Study population

Fifteen P-HIV (0/15 in Fiebig stage I; 4/15 in Fiebig stages II/III; 11/15 in Fiebig stages IV/V) and 13 C-HIV were studied. No differences were observed between groups in terms of demographics (Table 1), yet the former presented a significantly higher CD4^+^ T-cell nadir and a trend to higher CD4^+^ T-cell count at time of analysis as well as a lower prevalence of AIDS-defining events in their clinical history (Table 1).

**Table 1:** Epidemiological, clinical and immunological characteristics of the study groups.

	P-HIV (*n* = 15)	C-HIV (*n* = 13)	*P* value
Age (years), median (IQR)	42 (30.5–47.5)	41 (31–60)	0.52
Male sex [*n* (%)]	13 (87)	11 (85)	1
Risk factors for HIV infection			
Heterosexual [*n* (%)]	5 (33)	4 (31)	
MSM [*n* (%)]	9 (60)	6 (46)	0.20
IDU [*n* (%)]	1 (7)	0	
Other/unknown [*n* (%)]	0	3 (23)	
HBV/HCV co-infection [*n* (%)]	2 (13)	0	0.48
Nadir CD4^+^ count (cell/μl), median (IQR)	474 (330–576)	237 (50–422)	0.0074
CD4^+^ count (cell/μl), median (IQR)	518 (359–662)	378 (176–508)	0.060
CD4%, median (IQR)	25 (20–32)	17 (16–24)	0.067
CD8^+^ count (cell/mm^3^), median (IQR)	1060 (778–1532)	835 (717–1013)	0.17
CD8% at colonoscopy, median (IQR)	46 (42–60)	53 (48–64)	0.47
CD4^+^/CD8^+^ ratio, median (IQR)	0.67 (0.36–0.74)	0.34 (0.24–0.44)	0.12
HIV-RNA (log_10_ copies/ml) [median (IQR)]	5.1 (4.4–6.4)	5.4 (2.8–5.4)	0.19
Fiebig stage [*n* (%)]			
I	0	N/A	/
II/III	4 (27)	N/A	
IV–V	11 (73)	N/A	
AIDS-defining conditions or nadir CD4^+^ cell count <200 cells/μl [*n* (%)]	1 (7)	6 (46)	0.029

Data regarding continuous variables are presented as median (interquartile range; IQR), statistical analyses: unpaired *t* test, while data regarding discrete variables are presented as absolute numbers (%), statistical analyses: Fisher's exact test or chi-square test, as appropriate. C-HIV, people with chronic HIV infection; HBV, hepatitis B virus; IDU, intravenous drug use; IQR, interquartile range; N/A, not applicable; P-HIV, people with primary HIV infection.

### People with primary HIV infection show higher HIV DNA levels in colon biopsies

**Fig. 1:**
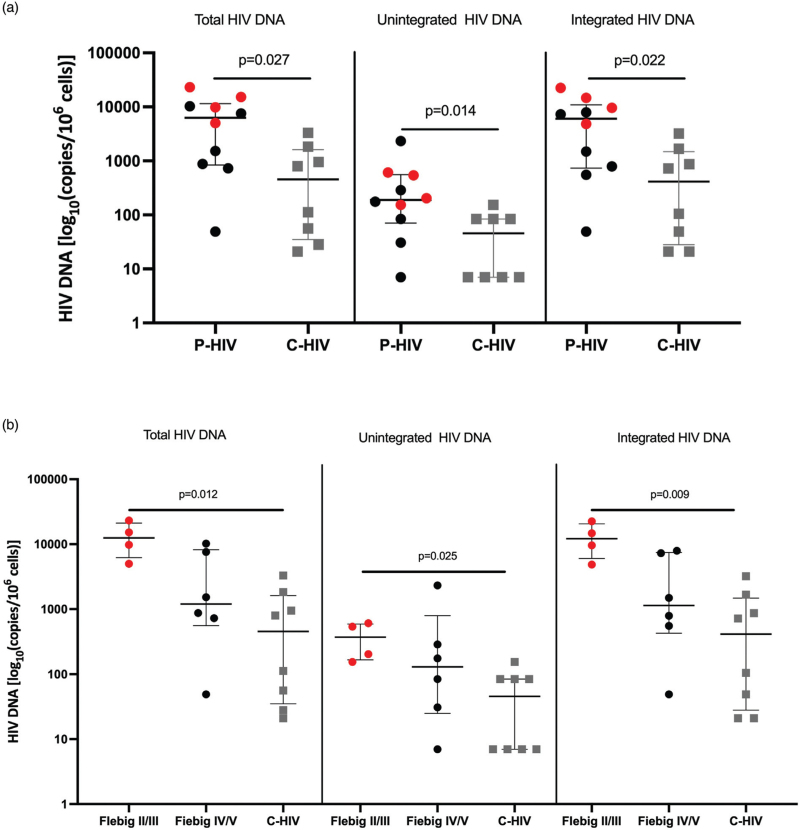
HIV DNA quantification in gut biopsies.

Significantly higher gut HIV DNA was found in P-HIV compared with C-HIV, with 6290 copies/10^6^ cells (IQR 838.3–11480) in the former and 455 copies/10^6^ cells (IQR 35–1614) in the latter (*P* = 0.027; Fig. 1a). P-HIV also presented higher levels of unintegrated HIV-DNA [189 copies/10^6^ cells (IQR 70.70–556.5) compared with C-HIV [45 copies/10^6^ cells (IQR 7–84)] (*P* = 0.014) as well as integrated HIV DNA [6069 copies/10^6^ cells (IQR 731.5–10862) vs. 413 copies/10^6^ cells (IQR 28–1477), *P* = 0.022] (Fig. 1a).

HIV DNA copies per 10^6^ cells were quantified in gut biopsies from P-HIV (*n* = 10) and C-HIV (*n* = 8) (a) and according to Fiebig stage (b; Fiebig II–III, *n* = 4; Fiebig IV–V, *n* = 6). Red dots indicate P-HIV individuals in Fiebig stages II–III.

Of note, a hierarchal distribution of HIV reservoirs was found in colon tissue according to the duration of HIV infection, with the highest levels of total, unintegrated and integrated HIV DNA in P-HIV in early Fiebig stages (II/III) followed by P-HIV in later Fiebig stages (IV/V) and, finally, C-HIV (Fig. 1b). In this respect, the comparison between P-HIV in Fiebig II/III and C-HIV was the only one to result in statistical significance for all HIV DNA measures [total HIV DNA: 12 513 copies/μg (IQR 6200–21 130) vs. 455 copies/μg (IQR 35–1614); *P* = 0.012]; unintegrated HIV DNA: 371 copies/μg (IQR 166.3–591.3) vs. 45.50 copies/ug (IQR 7–84); *P* = 0.025; integrated HIV DNA: 12 142 copies/μg (IQR 6034–20538) vs. 413 copies/μg (IQR 28–1477); *P* = 0.009] (Fig. 1b).

### People with primary HIV infection and people with chronic HIV feature comparable fibrosis, CD4^+^ T-cell depletion and neutrophil infiltration in colon biopsies

**Fig. 2:**
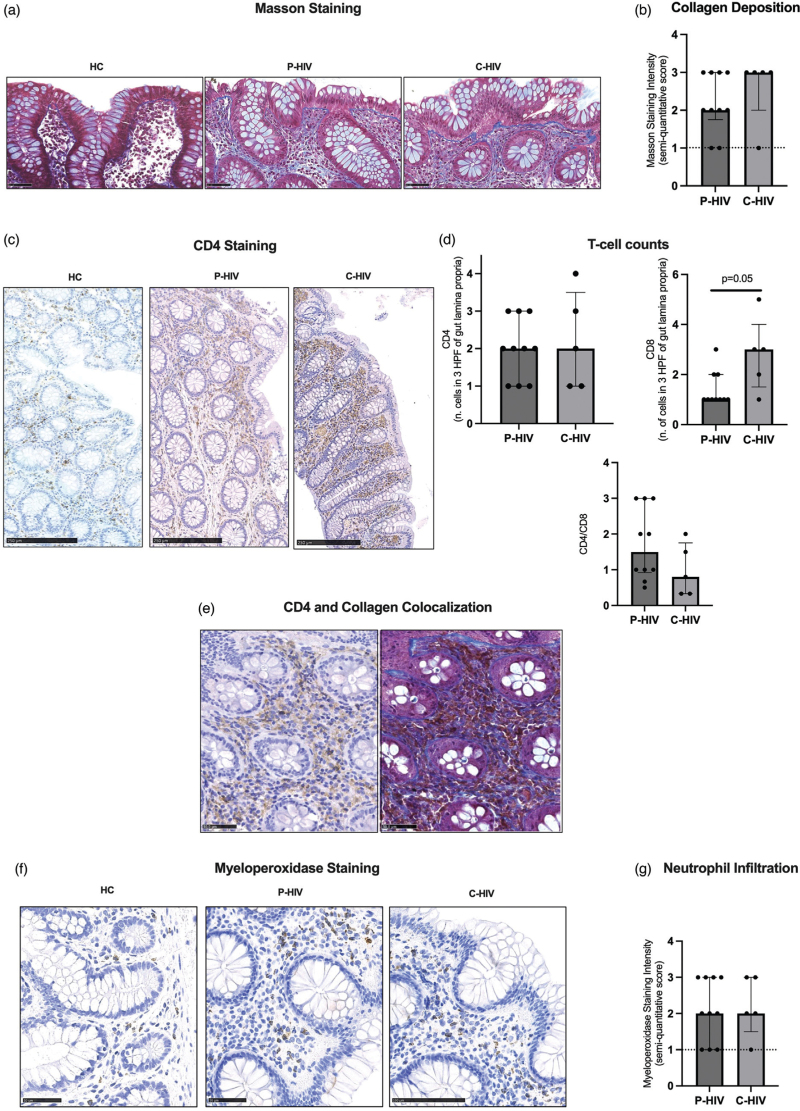
Collagen deposition, T-cell counts and neutrophil infiltration in gut biopsies.

Having shown higher HIV DNA in the gut of P-HIV compared with C-HIV, we assessed whether the two groups differed in terms of fibrosis, CD4^+^ T-cell depletion and neutrophil infiltration at mucosal sites. Collagen deposition in the colon of P-HIV was similar to that detected in C-HIV [2, (IQR 1.75–3) vs. 3 (IQR 2–3); *P* = 0.3; Fig. 2a and b) as was the mucosal CD4^+^ T-cell count [P-HIV: 2, (IQR 1–3); C-HIV: 2 (IQR 1–3.4); *P* = 0.8; Fig. 2c and d). We also found CD4^+^ and collagen colocalization (Fig. 2e).

Representative sections from lamina propria of healthy control (HC, left), P-HIV (middle) and (C-HIV) (right) were stained with trichrome to identify collagen fibres (a). Collagen deposition was higher in the GALT of HIV-infected, compared with HC individuals. Magnitude of fibrosis (blue staining intensity) was determined by using a semiquantitative score and the mean percentage area that stained positive is shown in B (P-HIV, *n* = 10; C-HIV, *n* = 5). The dotted line indicates the median value in representative HIV-uninfected controls (*n* = 5). Sections of colon biopsy from HC (left) and P-HIV (middle) and C-HIV (right) individuals were stained with antibodies against CD4^+^ and images were captured for quantitative image analysis (c). CD4^+^ and CD8^+^ T lymphocytes were determined by number of cells in 3 HPF of gut lamina propria; the CD4^+^/CD8^+^ ratio was calculated by dividing CD4^+^ by CD8^+^ (d) (P-HIV, *n* = 10; C-HIV, *n* = 5). Representative zoom showing CD4^+^ (left, brown) and collagen colocalization (right, blue) in a representative C-HIV (e). Representative sections from lamina propria of HC (left), P-HIV (middle) and (C-HIV) (right) were stained to detect neutrophil infiltration by myeloperoxidase (MPO) (f). Magnitude of neutrophil infiltration (brown staining intensity) was determined by using a semiquantitative score and the mean percentage area that stained positive is shown in G (P-HIV, *n* = 10; C-HIV, *n* = 5). The dotted line indicates the median value in representative HIV-uninfected controls (*n* = 5).

Of note, P-HIV displayed a trend to lower CD8^+^ T cells [1, IQR (1–2) vs. 3 (1.5–4); *P* = 0.05 and higher CD4^+^/CD8^+^ ratio [1.5 (1–3) vs. 0.8 (0.3–1.75); *P* = 0.15] in colon tissue compared with C-HIV (Fig. 2d).

We next measured the degree of neutrophil infiltration in colon and found no differences in MPO staining between P-HIV and C-HIV (Fig. 2f and g).

### Higher colon CDh-1 expression in people with primary HIV infection than people with chronic HIV

Having shown a similar degree of neutrophil infiltration and collagen deposition in the colonic mucosa of P-HIV and C-HIV, we next investigated gut barrier integrity through an IHC study of junctional complex proteins (CDh-1 and ZO-1).

**Fig. 3:**
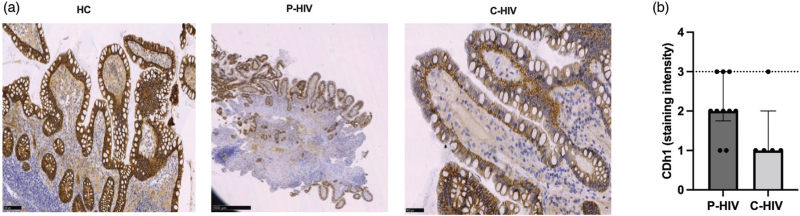
Tight junction protein expression in colon tissue of people with primary HIV and chronic HIV.

Both P-HIV and C-HIV displayed CDh-1 and ZO-1 protein expression preferentially located in lateral and basal zones with respect to controls (Fig. 3a). However, a trend to higher lateral and baso-lateral CDh-1 expression and greater staining intensity was observed in P-HIV compared with C-HIV (*P* = 0.12; Fig. 3b).

Representative sections from lamina propria of a heathy control (HC, left), P-HIV (middle) and C-HIV (right) were stained to identify E-cadherin (Cdh1, brown) (a). The mean percentage area that stained positive for Cdh1 is shown in B (P-HIV, *n* = 10; C-HIV, *df* = 5). The dotted line indicates the median value in representative HIV-uninfected controls (n = 5).

No significant differences were found between groups in terms of ZO-1 expression (data not shown).

### Microbial translocation is contained during acute HIV infection

We then asked whether the partial preservation of JCs proteins in P-HIV translated into the containment of microbial translocation from the gut lumen to the systemic circulation.

**Fig. 4:**
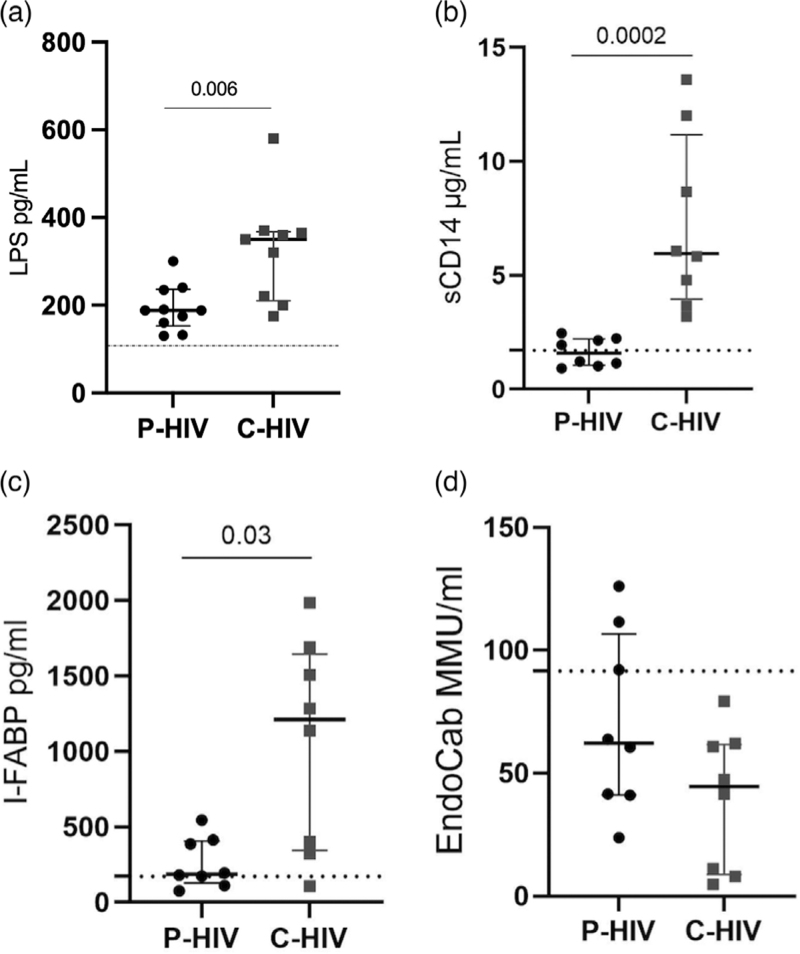
Markers of microbial translocation and intestinal damage in plasma.

Overall, measures of microbial translocation and intestinal barrier impairment were lower in P-HIV compared with C-HIV. Indeed, the former displayed significantly lower LPS [188.8 pg/ml (IQR 145.1–234.2) vs. 345.8 pg/ml (IQR 216.4–368.3), *P* = 0.006] (Fig. 4a), sCD14 [1.58 μg/ml (IQR 1.05–2.208) vs. 5.953 μg/ml (IQR 3.95–11.17), *P* = 0.0002] (Fig. 4b) and I-FABP [186.9 pg/ml (IQR 127.7–406) vs. 1210 pg/ml (IQR 367.4–1644), *P* = 0.03] (Fig. 4c). No differences between groups were found in EndocAb levels (*P* = 0.1; Fig. 4d).

Markers of microbial translocation (LPS, sCD14, EndoCAb) and intestinal damage (I-FABP) were measured in plasma samples from P-HIV (*n* = 8) and C-HIV (*n* = 8) (a–d). The dotted line indicates median values in representative HIV-uninfected controls (*n* = 5).

No statistical correlation was found between plasma biomarkers (IFABP, EndocAb, sCD14) and gastrointestinal variables (collagen deposition, neutrophil infiltration, E-cadherin, mucosal immune subsets) (Supplemental Figure 2).

### Mucosal γδ T cells are linked with gut damage and microbial translocation

To further comprehend the possible correlates of preserved gut epithelial integrity and reduced microbial translocation in P-HIV, we lastly investigated a subset of mucosal-associated immune cells, Vδ1/Vδ2 γδ T cells.

A trend to higher gut total γδ T cells [P-HIV: 7.8 (IQR 6.4–10.0); C-HIV: 5.8 (IQR 4.8–7.0); *P* = 0.11)] (Supplemental Figure 3A) and Vδ1 cells [P-HIV: 3.56 (IQR 2.36–3.76); C-HIV: 2.95 (IQR 1.98–2.77); *P* = 0.28)] was observed in P-HIV (Supplemental Figure 3B), yet P-HIV and C-HIV displayed similar Vδ2 [P-HIV: 1.2 (IQR 1.0–1.6); C-HIV: 1.2 (IQR 1.0–1.7); *P* = 0.96] (Supplemental Figure 3C). Total γδ T cells and the Vδ1 subset inversely correlated with collagen deposition (*r* = −0.67, *P* = 0.06) and circulating sCD14 (*r* = −0.62, *P* = 0.09) (Supplemental Figure 2).

## Discussion

Gastrointestinal barrier damage is a pathogenic feature of HIV infection, leading to a ‘leaky gut’ with translocation of microbial bioproducts from the mucosal lumen to the systemic circulation, causing immune activation, which is a driver of disease progression in untreated disease [31]. Combination antiretroviral therapy (cART) only partially reverts such damage [10–12], and the ongoing alterations within the gastrointestinal tract continuously fuel microbial translocation and inflammation, thus contributing to noninfectious comorbidities and premature aging in virally suppressed individuals [8]. Further, despite significant decreases in total peripheral [32] and integrated gut HIV DNA following cART initiation in early Fiebig [33], HIV reservoirs persist in the gastrointestinal mucosa, making viral eradication impossible through antiviral treatment alone.

Understanding when the above-mentioned events precisely occur in the natural course of HIV infection, is of utmost importance in the best framing of HIV pathogenesis and HIV-associated comorbidities.

In the present work, we show higher tissue reservoirs in early HIV infection, yet similar neutrophil infiltration and collagen deposition to those observed in chronic HIV infection. In contrast, plasma microbial translocation markers were significantly lower in the former.

We first evaluated the HIV reservoir in P-HIV and C-HIV through the study of total, unintegrated and integrated HIV DNA forms. Total HIV DNA allows for the simultaneous quantification of all forms of HIV DNA in infected cells, which play a different role in HIV pathogenesis [34]. Indeed, the study of unintegrated and integrated HIV DNA provides insights into the dynamics and composition of the HIV reservoir [24,35–38]; however, these are not regarded as standard markers of the reservoir.

We report significantly higher HIV DNA levels in P-HIV compared with C-HIV. Given the normalization of HIV DNA copy numbers to 1 μg of cellular DNA and the finding of similar colon CD4^+^ T-cell counts in P-HIV and C-HIV, our results point to increased reservoirs in the earliest stages of acute HIV infection, which appear to be driven by individuals in Fiebig II/III. This is in partial disagreement with previous work demonstrating significantly lower gut reservoirs in the earliest Fiebig stage than later P-HIV stages/chronic infection [33] and may be explained by the absence of individuals in Fiebig I and the small sample size in our study. Of note, both studies show that the HIV reservoir in Fiebig IV/V resembles that of chronic HIV [33].

We then studied the expression of myeloperoxidase and Masson staining and show comparable colon neutrophil infiltration and fibrosis in C-HIV and P-HIV. Our IHC finding of collagen and CD4^+^ co-localization is in line with previous work revealing that the disruption of lymphoid tissue architecture is linked to CD4^+^ T-cell loss in all stages of HIV infection [39–41], as it suggests that gut fibrosis may be a contributing mechanism of CD4^+^ T-cell depletion. Of note, P-HIV displayed a trend to lower CD8^+^ and higher CD4^+^/CD8^+^ ratio in the gut, pointing to better gut mucosal immune competence.

HIV-associated gut dysfunction accounts for chronic low-grade inflammation, which is linked to metabolic and chronic diseases [42]. Although not equivocal in all studies [43], convincing evidence indicates a role for microbial translocation in disease progression in people with HIV (PWH). Indeed, sCD14 and I-FABP resulted independent predictors of all-cause mortality in treated HIV infection [44] and were associated with adiposity [45,46]; similarly, LPS predicted HIV disease progression in cART-naive individuals [47] and was linked to insulin resistance [48,49]. EndocAb have also been assessed in the study of HIV pathogenesis [31] as their levels increase as part of the normal humoral response to antigenic stimulation (i.e. gut-derived microbial products chronically present in the systemic circulation) [31].

Despite comparable gut lymphoid architecture, microbial translocation markers were significantly lower in P-HIV compared with C-HIV. This is in keeping with reported findings of lower LPS and 16 s rDNA in P-HIV [5, 17] and may reflect the timing of microbial translocation, which has been shown to occur in later stages of HIV/SIV infection [5,17,18]. Because the passage of microbial bioproducts from the gut to the systemic circulation is a consequence of increased mucosal permeability, we assessed whether differences in gut barrier integrity could explain our findings, demonstrating that the expression of E-cadherin, a protein of the gut junctional complex [50], was altered in HIV-infected individuals compared with uninfected controls, with a trend to higher expression in P-HIV than C-HIV. Thus, the reported hierarchical distribution of gut junctional proteins may partially explain why microbial translocation is controlled in primary HIV infection [17]. In addition, our findings shed light on the sequence of events in the HIV-driven pathogenic cascade showing that gut damage may follow reservoir establishment yet precede microbial translocation. It must be noted that Zonula Occludens 1 expression in the colon showed comparable levels in P-HIV than C-HIV, suggesting that gut barrier dysfunction is, tantamount to the other structural abnormalities described above, an extremely rapid-evolving process in primary HIV infection.

Mucosal immunity also plays a pivotal role in the regulation of gut barrier function and control over microbial translocation [2]. We hereby show that mucosal Vδ1 cells were lower in P-HIV compared with uninfected controls, yet higher than C-HIV, suggesting their progressive depletion during untreated HIV infection. Together with our results of a negative correlation between gut γδ T cells and sCD14/collagen, our findings suggest that these cell subsets may hinder microbial translocation through the maintenance of gut architecture.

Limitations of the present work include the exploratory nature of the study, because of the small sample size and the multiple comparisons performed without adjustment.

In conclusion, in primary HIV infection, we show increased tissue reservoirs, impairment of the gut–epithelial barrier and mucosal architecture with neutrophil infiltration, yet containment of microbial translocation, which may depend on the partial preservation of E-cadherin 1 and mucosal γδ T cells in early HIV infection.

## Acknowledgements

Author contributions: C.T. cared for and enrolled study participants, analysed and interpreted the data, designed the figures and wrote the manuscript. V.B. and E.S.C. analysed and interpreted the data, designed the figures and drafted the manuscript. D.T. and F.S. performed the experiments on gut tissue, analysed data and drafted the manuscript. C.F. and M.A. cared for and enrolled study participants. A.C. and C.O. performed HIV DNA on gut tissue. C.L. performed colonscopies. S.R., A.M., A.B., A.C., A.G. and S.N. coordinated the INACTION Study group and edited the manuscript. G.M. conceived and designed the study, interpreted the results and wrote the manuscript.

Italian Network of ACuTe HIV InfectiON (INACTION): Nicola Squillace (Monza); Giuseppe Tambussi, Marco Ripa, Raffele Dell’Acqua (H San Raffaele Milano); Andrea Antinori, Carmela Pinnetti (INMI Spallanzani Roma); Gianfranco Orofino, Ilaria De Benedetto, Micol Ferrara (Torino); Cristina Mussini, Vanni Borghi, Federica Carli (Modena); Benedetto Maurizio Celesia (Catania); Lucio Cosco, Carlo Torti (Catanzaro); Gabriella d’Ettorre (Umberto I Roma); Antonio Di Biagio (Genova); Emanuele Focà, Eugenia Quiros-Roland (Brescia); Antonina Franco (Siracusa); Diego Ripamonti, Franco Maggiolo (Bergamo); Roberto Gulminetti, Massimiliano Fabbiani (Pavia); Sandro Piga, Marzia Garau, Marco Campus (Cagliari); Tiziana Formenti, Sonia De Rose, Alessia Lai (H Sacco Milano); Antonella Cingolani (H Gemelli Roma); Giordano Madeddu (Sassari).

We thank all individuals who participated in the study and the staff of the Clinic of Infectious Diseases and Tropical Medicine at ‘ASST Santi Paolo e Carlo’ who cared for the patients.

The study was supported in part by the Italian Ministry of Health, grant ‘Ricerca Finalizzata’ (number NET-2013-02355333-3) to G.M.

Presented in part at CROI 2017, 13th–16th February 2017, Seattle, Washington, USA. Poster #272.

### Conflicts of interest

There are no conflicts of interest.

## Supplementary Material

Supplemental Digital Content

## References

[1] Nazli A Chan O Dobson-Belaire WN Ouellet M Tremblay MJ Gray-Owen SD. Exposure to HIV-1 directly impairs mucosal epithelial barrier integrity allowing microbial translocation. *PLoS Pathog* 2010; 6:e1000852. 20386714 10.1371/journal.ppat.1000852 PMC2851733

[2] Veazey RS. Intestinal CD4 depletion in HIV/SIV infection. *Curr Immunol Rev* 2019; 15:76–91. 31431807 10.2174/1573395514666180605083448 PMC6701936

[3] Klatt NR Estes JD Sun X Ortiz AM Barber JS Harris LD. Loss of mucosal CD103+ DCs and IL-17+ and IL-22+ lymphocytes is associated with mucosal damage in SIV infection. *Mucosal Immunol* 2012; 5:646–657. 22643849 10.1038/mi.2012.38 PMC3443541

[4] Walker EM Slisarenko N Gerrets GL Grasperge BF Mattison JA Kissinger PJ. Dysregulation of IL-17/IL-22 effector functions in blood and gut mucosal gamma delta T cells correlates with increase in circulating leaky gut and inflammatory markers during cART-treated chronic SIV infection in macaques. *Front Immunol* 2021; 12:647398. 33717202 10.3389/fimmu.2021.647398 PMC7946846

[5] Brenchley JM Price DA Schacker TW Asher TE Silvestri G Rao S. Microbial translocation is a cause of systemic immune activation in chronic HIV infection. *Nat Med* 2006; 12:1365–1371. 17115046 10.1038/nm1511

[6] Cecchinato V Trindade CJ Laurence A Heraud JM Brenchley JM Ferrari MG. Altered balance between Th17 and Th1 cells at mucosal sites predicts AIDS progression in simian immunodeficiency virus-infected macaques. *Mucosal Immunol* 2008; 1:279–288. 19079189 10.1038/mi.2008.14 PMC2997489

[7] Canary LA Vinton CL Morcock DR Pierce JB Estes JD Brenchley JM Klatt NR. Rate of AIDS progression is associated with gastrointestinal dysfunction in simian immunodeficiency virus-infected pigtail macaques. *J Immunol* 2013; 190:2959–2965. 23401593 10.4049/jimmunol.1202319 PMC3665608

[8] Hunt PW Sinclair E Rodriguez B Shive C Clagett B Funderburg N. Gut epithelial barrier dysfunction and innate immune activation predict mortality in treated HIV infection. *J Infect Dis* 2014; 210:1228–1238. 24755434 10.1093/infdis/jiu238 PMC4192038

[9] Hunt PW Cao HL Muzoora C Ssewanyana I Bennett J Emenyonu N. Impact of CD8+ T-cell activation on CD4+ T-cell recovery and mortality in HIV-infected Ugandans initiating antiretroviral therapy. *AIDS* 2011; 25:2123–2131. 21881481 10.1097/QAD.0b013e32834c4ac1 PMC3480326

[10] Somsouk M Estes JD Deleage C Dunham RM Albright R Inadomi JM. Gut epithelial barrier and systemic inflammation during chronic HIV infection. *AIDS* 2015; 29:43–51. 25387317 10.1097/QAD.0000000000000511 PMC4444362

[11] Tincati C Merlini E Braidotti P Ancona G Savi F Tosi D. Impaired gut junctional complexes feature late-treated individuals with suboptimal CD4+ T-cell recovery upon virologically suppressive combination antiretroviral therapy. *AIDS* 2016; 30:991–1003. 27028142 10.1097/QAD.0000000000001015

[12] Chung CY Alden SL Funderburg NT Fu P Levine AD. Progressive proximal-to-distal reduction in expression of the tight junction complex in colonic epithelium of virally-suppressed HIV+ individuals. *PLoS Pathog* 2014; 10:e1004198. 24968145 10.1371/journal.ppat.1004198 PMC4072797

[13] Nganou-Makamdop K Talla A Sharma AA Darko S Ransier A Laboune F. Translocated microbiome composition determines immunological outcome in treated HIV infection. *Cell* 2021; 184:3899.e16–3914.e16. 34237254 10.1016/j.cell.2021.05.023 PMC8316372

[14] Bono V Augello M Tincati C Marchetti G. Failure of CD4+ T-cell recovery upon virally-effective cART: an enduring gap in the understanding of HIV+ immunological non-responders. *New Microbiol* 2022; 45:155–172. 35920870

[15] Brenchley JM Schacker TW Ruff LE Price DA Taylor JH Beilman GJ. CD4+ T cell depletion during all stages of HIV disease occurs predominantly in the gastrointestinal tract. *J Exp Med* 2004; 200:749–759. 15365096 10.1084/jem.20040874 PMC2211962

[16] Mattapallil JJ Douek DC Hill B Nishimura Y Martin M Roederer M. Massive infection and loss of memory CD4+ T cells in multiple tissues during acute SIV infection. *Nature* 2005; 434:1093–1097. 15793563 10.1038/nature03501

[17] Chevalier MF Petitjean G Dunyach-Rémy C Didier C Girard PM Manea ME. The Th17/Treg ratio, IL-1RA and sCD14 levels in primary HIV infection predict the T-cell activation set point in the absence of systemic microbial translocation. *PLoS Pathog* 2013; 9:e1003453. 23818854 10.1371/journal.ppat.1003453 PMC3688532

[18] Hensley-McBain T Berard AR Manuzak JA Miller CJ Zevin AS Polacino P. Intestinal damage precedes mucosal immune dysfunction in SIV infection. *Mucosal Immunol* 2018; 11:1429–1440. 29907866 10.1038/s41385-018-0032-5 PMC6162106

[19] Schuetz A Deleage C Sereti I Rerknimitr R Phanuphak N Phuang-Ngern Y. RV254/SEARCH 010 and RV304/SEARCH 013 Study Groups. Initiation of ART during early acute HIV infection preserves mucosal Th17 function and reverses HIV-related immune activation. *PLoS Pathog* 2014; 10:e1004543. 25503054 10.1371/journal.ppat.1004543 PMC4263756

[20] Berard AR Hensley-McBain T Noël-Romas L Birse K Abou M Westmacott G. Mass spectrometry analysis of gut tissue in acute SIV-infection in rhesus macaques identifies early proteome alterations preceding the interferon inflammatory response. *Sci Rep* 2023; 13:690. 36639424 10.1038/s41598-022-27112-y PMC9839751

[21] Fiebig EW Wright DJ Rawal BD Garrett PE Schumacher RT Peddada L. Dynamics of HIV viremia and antibody seroconversion in plasma donors: implications for diagnosis and staging of primary HIV infection. *AIDS* 2003; 17:1871–1879. 12960819 10.1097/00002030-200309050-00005

[22] Hart AL Al-Hassi HO Rigby RJ Bell SJ Emmanuel AV Knight SC. Characteristics of intestinal dendritic cells in inflammatory bowel diseases. *Gastroenterology* 2005; 129:50–65. 16012934 10.1053/j.gastro.2005.05.013

[23] Casabianca A Orlandi C Canovari B Scotti M Acetoso M Valentini M. A real time PCR platform for the simultaneous quantification of total and extrachromosomal HIV DNA forms in blood of HIV-1 infected patients. *PLoS One* 2014; 9:e111919. 25364909 10.1371/journal.pone.0111919 PMC4218859

[24] Orlandi C Canovari B Bozzano F Marras F Pasquini Z Barchiesi F. A comparative analysis of unintegrated HIV-1 DNA measurement as a potential biomarker of the cellular reservoir in the blood of patients controlling and noncontrolling viral replication. *J Transl Med* 2020; 18:204. 32429953 10.1186/s12967-020-02368-y PMC7236182

[25] Cannizzo ES Bellistrì GM Casabianca A Tincati C Iannotti N Barco A. Immunophenotype and function of CD38-expressing CD4+ and CD8+ T cells in HIV-infected patients undergoing suppressive combination antiretroviral therapy. *J Infect Dis* 2015; 211:1511–1513. 25398457 10.1093/infdis/jiu634

[26] Marras F Casabianca A Bozzano F Ascierto ML Orlandi C Di Biagio A. Control of the HIV-1 DNA reservoir is associated. *J Virol* 2017; 91:e00647-17. 10.1128/JVI.00647-17 PMC5686752 28956765

[27] Surdo M Cortese MF Orlandi C Di Santo F Aquaro S Magnani M. Different kinetics of viral replication and DNA integration in the main HIV-1 cellular reservoirs in the presence and absence of integrase inhibitors. *Antiviral Res* 2018; 160:165–174. 30420339 10.1016/j.antiviral.2018.10.017

[28] Merlini E Cazzaniga FA Casabianca A Orlandi C Magnani M Ancona G. Reduction of immune activation and partial recovery of Staphylococcal enterotoxin B-induced cytokine production after switching to an integrase strand transfer inhibitor-containing regimen: results from an observational cohort study. *Clin Drug Investig* 2019; 39:1239–1249. 10.1007/s40261-019-00840-2 PMC6842342 31531832

[29] Taramasso L Bozzano F Casabianca A Orlandi C Bovis F Mora S. Persistence of unintegrated HIV DNA associates with ongoing NK cell activation and CD34+DNAM-1brightCXCR4+ precursor turnover in vertically infected patients despite successful antiretroviral treatment. *Front Immunol* 2022; 13:847816. 35558085 10.3389/fimmu.2022.847816 PMC9088003

[30] Gregory TR. Animal genome size database In; 2023.

[31] Marchetti G Tincati C Silvestri G. Microbial translocation in the pathogenesis of HIV infection and AIDS. *Clin Microbiol Rev* 2013; 26:2–18. 23297256 10.1128/CMR.00050-12 PMC3553668

[32] Bruzzesi E Gabrieli A Bernasconi D Marchetti G Calcagno A Ripamonti D. INACTION Study Group. HIV-DNA decrease during treatment in primary HIV-1 infection with three different drug regimens: Italian Network of Acute HIV Infection (INACTION) clinical trial. *J Med Virol* 2023; 95:e29114. 37752816 10.1002/jmv.29114

[33] Leyre L Kroon E Vandergeeten C Sacdalan C Colby DJ Buranapraditkun S. Abundant HIV-infected cells in blood and tissues are rapidly cleared upon ART initiation during acute HIV infection. *Sci Transl Med* 2020; 12:eaav3491. 32132218 10.1126/scitranslmed.aav3491 PMC7293182

[34] Avettand-Fènoël V Hocqueloux L Ghosn J Cheret A Frange P Melard A. Total HIV-1 DNA, a marker of viral reservoir dynamics with clinical implications. *Clin Microbiol Rev* 2016; 29:859–880. 27559075 10.1128/CMR.00015-16 PMC5010749

[35] Koelsch KK Liu L Haubrich R May S Havlir D Günthard HF. Dynamics of total, linear nonintegrated, and integrated HIV-1 DNA in vivo and in vitro. *J Infect Dis* 2008; 197:411–419. 18248304 10.1086/525283

[36] Suspène R Meyerhans A. Quantification of unintegrated HIV-1 DNA at the single cell level in vivo. *PLoS One* 2012; 7:e36246. 22574142 10.1371/journal.pone.0036246 PMC3344866

[37] Mexas AM Graf EH Pace MJ Yu JJ Papasavvas E Azzoni L. Concurrent measures of total and integrated HIV DNA monitor reservoirs and ongoing replication in eradication trials. *AIDS* 2012; 26:2295–2306. 23014521 10.1097/QAD.0b013e32835a5c2f PMC4692807

[38] Agosto LM Liszewski MK Mexas A Graf E Pace M Yu JJ. Patients on HAART often have an excess of unintegrated HIV DNA: implications for monitoring reservoirs. *Virology* 2011; 409:46–53. 20970154 10.1016/j.virol.2010.08.024 PMC3253773

[39] Zeng M Smith AJ Wietgrefe SW Southern PJ Schacker TW Reilly CS. Cumulative mechanisms of lymphoid tissue fibrosis and T cell depletion in HIV-1 and SIV infections. *J Clin Invest* 2011; 121:998–1008. 21393864 10.1172/JCI45157 PMC3049394

[40] Estes JD Wietgrefe S Schacker T Southern P Beilman G Reilly C. Simian immunodeficiency virus-induced lymphatic tissue fibrosis is mediated by transforming growth factor beta 1-positive regulatory T cells and begins in early infection. *J Infect Dis* 2007; 195:551–561. 17230415 10.1086/510852

[41] Schacker TW Brenchley JM Beilman GJ Reilly C Pambuccian SE Taylor J. Lymphatic tissue fibrosis is associated with reduced numbers of naive CD4+ T cells in human immunodeficiency virus type 1 infection. *Clin Vaccine Immunol* 2006; 13:556–560. 16682476 10.1128/CVI.13.5.556-560.2006 PMC1459657

[42] Sim JH Mukerji SS Russo SC Lo J. Gastrointestinal dysfunction and HIV comorbidities. *Curr HIV/AIDS Rep* 2021; 18:57–62. 33469815 10.1007/s11904-020-00537-8 PMC8530437

[43] Merlini E Cozzi-Lepri A Castagna A Costantini A Caputo SL Carrara S. Inflammation and microbial translocation measured prior to combination antiretroviral therapy (cART) and long-term probability of clinical progression in people living with HIV. *BMC Infect Dis* 2021; 21:557. 34116650 10.1186/s12879-021-06260-y PMC8196504

[44] Sandler NG Wand H Roque A Law M Nason MC Nixon DE. INSIGHT SMART Study Group. Plasma levels of soluble CD14 independently predict mortality in HIV infection. *J Infect Dis* 2011; 203:780–790. 21252259 10.1093/infdis/jiq118 PMC3071127

[45] Mave V Erlandson KM Gupte N Balagopal A Asmuth DM Campbell TB. ACTG PEARLS and NWCS 319 Study Team. Inflammation and change in body weight with antiretroviral therapy initiation in a multinational cohort of HIV-infected adults. *J Infect Dis* 2016; 214:65–72. 26962236 10.1093/infdis/jiw096 PMC4907416

[46] El Kamari V Moser C Hileman CO Currier JS Brown TT Johnston L. Lower pretreatment gut integrity is independently associated with fat gain on antiretroviral therapy. *Clin Infect Dis* 2019; 68:1394–1401. 30137242 10.1093/cid/ciy716 PMC6599164

[47] Marchetti G Cozzi-Lepri A Merlini E Bellistrì GM Castagna A Galli M. ICONA Foundation Study Group. Microbial translocation predicts disease progression of HIV-infected antiretroviral-naive patients with high CD4+ cell count. *AIDS* 2011; 25:1385–1394. 21505312 10.1097/QAD.0b013e3283471d10

[48] Pedersen KK Pedersen M Trøseid M Gaardbo JC Lund TT Thomsen C. Microbial translocation in HIV infection is associated with dyslipidemia, insulin resistance, and risk of myocardial infarction. *J Acquir Immune Defic Syndr* 2013; 64:425–433. 23797689 10.1097/QAI.0b013e31829f919d

[49] Timmons T Shen C Aldrovandi G Rollie A Gupta SK Stein JH Dubé MP. Microbial translocation and metabolic and body composition measures in treated and untreated HIV infection. *AIDS Res Hum Retroviruses* 2014; 30:272–277. 24033288 10.1089/aid.2013.0162 PMC3938948

[50] Hartsock A Nelson WJ. Adherens and tight junctions: structure, function and connections to the actin cytoskeleton. *Biochim Biophys Acta* 2008; 1778:660–669. 17854762 10.1016/j.bbamem.2007.07.012 PMC2682436

