# Supplementary material for: Primary HIV infection features colonic damage and neutrophil inflammation yet containment of microbial translocation
Source: AIDS. 2023 Dec 4;38(5):623–32. doi: 10.1097/QAD.0000000000003799 (PMC10942218; doi:10.1097/QAD.0000000000003799)
Supplement: Supplemental Digital Content [file aids-38-623-s001.pdf]

Primary HIV Infection Features Colonic Damage and Neutrophil Inflammation yet Containment of Microbial Translocation

Supplemental Digital Content

Supplementary Figure 1. Gating strategy for γδ T-cells in colon tissue

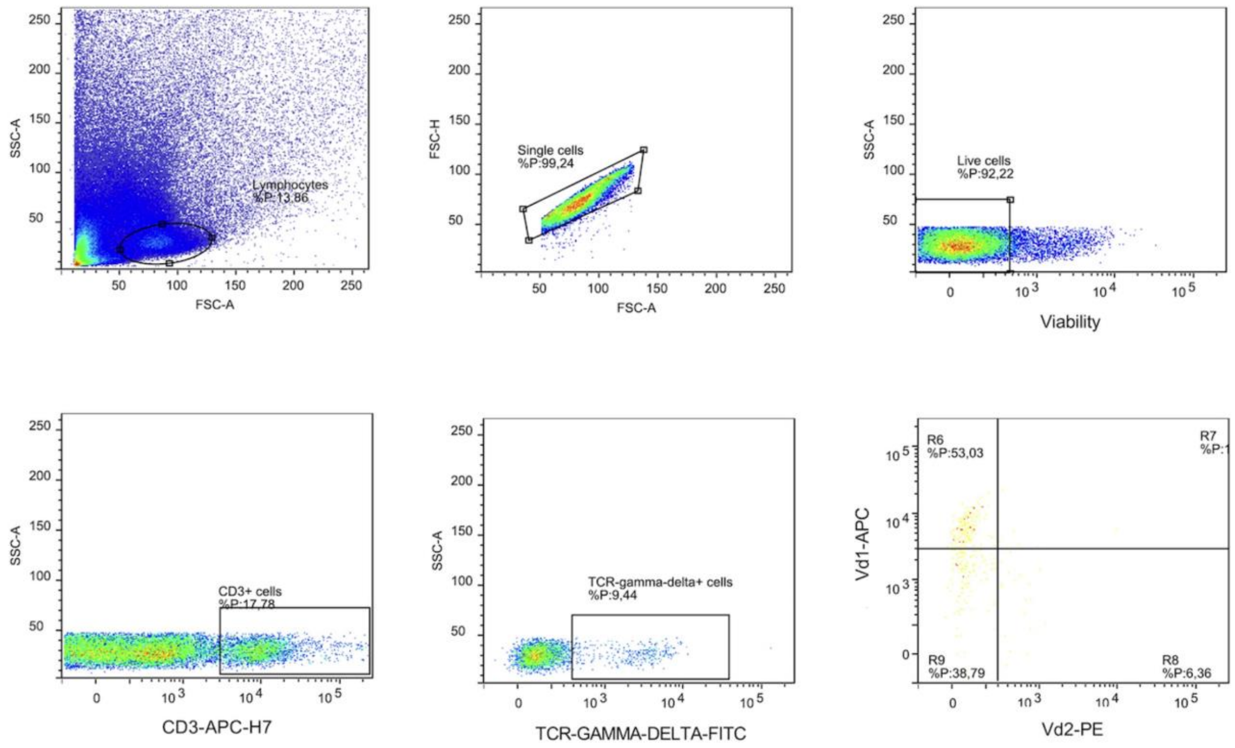

Supplemental Figure 2. Correlations between plasma biomarkers and gastrointestinal variables

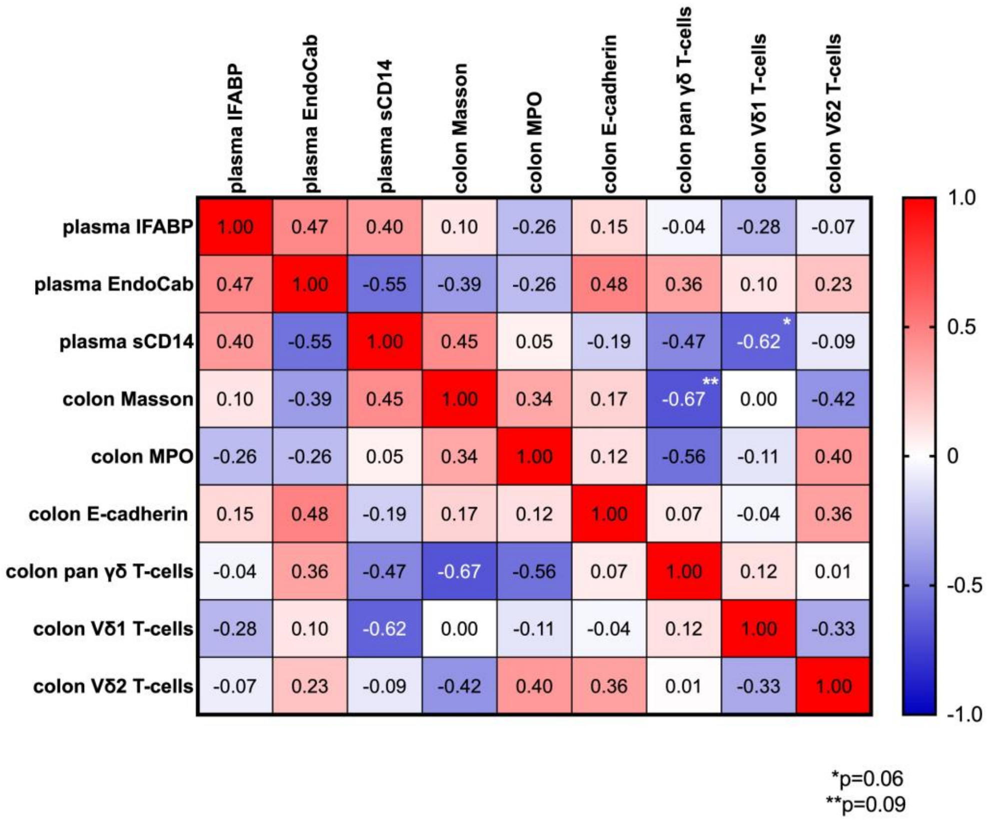

Correlations were evaluated by Spearman's correlation. R-values are presented in the figure. P-values with a trend to statistical significance are indicated with * and **.

Supplemental Figure 3. Mucosal γδ T-cell substes in P-HIV and C-HIV

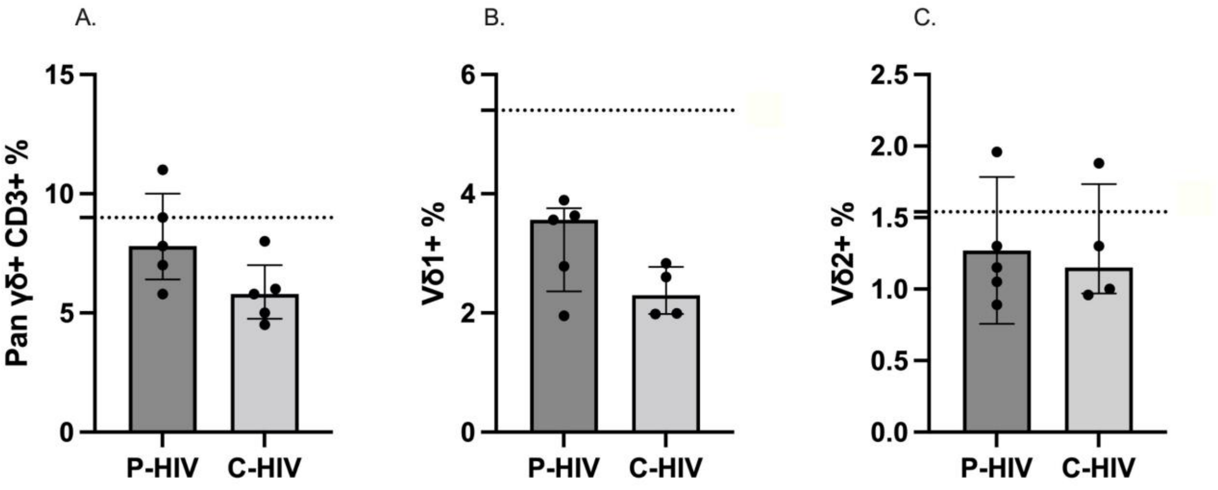

Total γδ T-cell (A), Vδ1 (B) and Vδ2 (C) subsets were measured by flow cytometry in P-HIV (n=5) and C-HIV (n=4). The dotted line indicates median values in representative samples from HIV-uninfected controls (n=4).
